# NF-κB/mTOR/MYC Axis Drives PRMT5 Protein Induction After T Cell Activation via Transcriptional and Non-transcriptional Mechanisms

**DOI:** 10.3389/fimmu.2019.00524

**Published:** 2019-03-19

**Authors:** Lindsay M. Webb, Janiret Narvaez Miranda, Stephanie A. Amici, Shouvonik Sengupta, Gregory Nagy, Mireia Guerau-de-Arellano

**Affiliations:** ^1^Division of Medical Laboratory Science, Wexner Medical Center, School of Health and Rehabilitation Sciences, College of Medicine, The Ohio State University, Columbus, OH, United States; ^2^Biomedical Sciences Graduate Program, The Ohio State University, Columbus, OH, United States; ^3^Institute for Behavioral Medicine Research, The Ohio State University, Columbus, OH, United States; ^4^Department of Microbial Infection and Immunity, The Ohio State University, Columbus, OH, United States; ^5^Department of Neuroscience, The Ohio State University, Columbus, OH, United States

**Keywords:** PRMT5, T cell, signaling, multiple sclerosis, naive, memory

## Abstract

Multiple sclerosis is an autoimmune disease of the central nervous system (CNS) mediated by CD4^+^ T cells and modeled via experimental autoimmune encephalomyelitis (EAE). Inhibition of PRMT5, the major Type II arginine methyltransferase, suppresses pathogenic T cell responses and EAE. PRMT5 is transiently induced in proliferating memory inflammatory Th1 cells and during EAE. However, the mechanisms driving PRMT5 protein induction and repression as T cells expand and return to resting is currently unknown. Here, we used naive mouse and memory mouse and human Th1/Th2 cells as models to identify mechanisms controlling PRMT5 protein expression in initial and recall T cell activation. Initial activation of naive mouse T cells resulted in NF-κB-dependent transient *Prmt5* transcription and NF-κB, mTOR and MYC-dependent PRMT5 protein induction. In murine memory Th cells, transcription and miRNA loss supported PRMT5 induction to a lesser extent than in naive T cells. In contrast, NF-κB/MYC/mTOR-dependent non-transcriptional PRMT5 induction played a major role. These results highlight the importance of the NF-κB/mTOR/MYC axis in PRMT5-driven pathogenic T cell expansion and may guide targeted therapeutic strategies for MS.

## Introduction

Multiple sclerosis (MS) is an inflammatory disease of the central nervous system (CNS), thought to be driven by myelin-reactive inflammatory T cells. T cell responses against myelin basic protein (MBP), myelin oligodendrocyte glycoprotein (MOG), and proteolipid protein (PLP) antigens are thought to underlie MS. In support of this notion, immunization against these antigens induces the MS-like disease experimental autoimmune encephalomyelitis (EAE) in mice and other rodents (1–3). MS and EAE are thought to be driven by an imbalance in inflammatory and regulatory T cell responses. Increased pathogenic, myelin-reactive T helper (Th) 1 and Th17 responses drive demyelination and disability (4), while beneficial Th2 and regulatory T (Treg) cell responses are deficient in MS patients (5). MS active disease and development of relapses in MS patients has been linked to the reactivation and expansion of pathogenic inflammatory Th cells (6–9). These findings highlight the importance of understanding mechanisms driving pathogenic Th cell expansion.

We recently reported that protein arginine methyltransferase 5 (PRMT5) plays a crucial role in inflammatory T cell expansion and EAE disease (10). PRMT5 is a type II arginine methyltransferase that catalyzes symmetric dimethylation (SDM) of arginine on histones and other proteins (11–13). PRMT5 has long been known as an epigenetic modifier and regulator of gene expression and has a well-established role in development (12, 14), hematopoiesis (15), and cancer (12, 16). In contrast, the role of PRMT5 in T cells and autoimmunity is a relatively new field of study. PRMT5 induction is observed in lymphoid organs after immunization, just prior to the development of EAE signs (10). PRMT5 protein induction was also observed after activation of isolated T cells, and is conserved in both mouse and human recall memory Th cell responses. Modulating PRMT5 activity with selective PRMT5 inhibitors preferentially suppresses inflammatory Th1 vs. Th2 cell proliferation and EAE, indicating that PRMT5 may be a therapeutic target in MS (10). However, the mechanisms that drive PRMT5 expression during initial and recall T cell activation are currently unknown.

Some clues into drivers and mechanisms regulating PRMT5 protein expression may be extrapolated from cancer cells and models, in which PRMT5 protein is generally induced and contributes to tumorigenicity (17, 18). First, microRNAs (miRNAs) were shown to regulate PRMT5 protein expression via a non-transcriptional mechanism, namely translational repression. Loss of miR-96 and miR-92b in mantle cell lymphoma cell lines led to uncontrolled PRMT5 protein translation and lymphoma cell proliferation (19, 20). Loss of *PRMT5*-targeting miR-96 and miR-92b in Epstein Barr Virus-transformed cell lines was driven by nuclear factor (NF)-κB signaling and resulted in PRMT5 protein induction (21). MYC is another known PRMT5 regulator. Specifically, MYC has been shown to promote *PRMT5* mRNA transcription in B cell lymphoma (22, 23). A remaining question is whether PRMT5 expression is similarly regulated in T cells and whether these mechanisms differ between naive vs. memory T cells and/or between mouse and human T cells.

TcR stimulation induces drastic alterations in gene expression through the activation of multiple highly regulated signaling pathways, including NF-κB, extracellular signal-regulated kinase (Erk), phosphoinositide 3-kinase (PI3K), and mammalian target of rapamycin (mTOR) pathways. The Erk pathway drives transcription and translocation of transcription factor Fos into the nucleus, which together with Jun, forms the functional Activator Protein 1 (AP-1) complex. Transcription factors NF-κB and AP-1 converge to rapidly upregulate IL-2 expression, a growth, and survival cytokine that drives T cell expansion (24, 25). PI3K/mTOR activation promotes protein translation, which together with MYC pathway regulate the metabolic shift to glycolysis, in order to meet the biosynthetic demands of growing and dividing T cells (26–28). MYC induction is also essential for driving T cell activation and proliferation (29). Given that the integrated signals of the TcR signaling network control the magnitude of T cell division and effector functions, excessive or dysregulated TcR signaling could lead to loss of immune tolerance and autoimmunity (30–37). For instance, there is evidence that MS patients' T cells display an activated or memory phenotype (38, 39), even though circulating myelin-specific T cells exist in both healthy individuals and MS patients (40, 41). Similarly, genome-wide association studies (GWAS) in MS patients have identified single nucleotide polymorphisms (SNPs) linked to the *MYC* and NF-κB complex genes, implicating TcR signaling pathways in MS (42–44). In addition, NF-κB signaling is overactive in MS patients and certain MS-risk NF-κB complex SNPs increase NF-κB signaling in T cells (44, 45). Given the links between NF-κB/MYC signaling and PRMT5 induction in cancer (21, 22) as well as between NF-κB/MYC and MS, it is important to investigate the impact of these pathways in T cell PRMT5 expression and pathogenic T cell responses.

In this study, we explore the signaling pathways and mechanisms driving PRMT5 expression after T cell activation. Using murine naive and memory as well as human memory Th cells as models of initial and recall T cell activation, we show that PRMT5 protein expression is regulated via a combination of transcriptional and non-transcriptional mechanisms. NF-κB, mTOR and MYC pathways promoted PRMT5 protein induction in murine naive and memory T cells. However, some differences in the mechanisms of PRMT5 regulation were observed between naive and memory T cells. In naive T cells, NF-κB induced both *Prmt5* transcription and PRMT5 protein induction, the latter mediated by MYC induction and mTOR-induced miR-322 loss. In contrast, in memory Th cells, the NF-κB/MYC/mTOR axis was dispensable for *Prmt5* transcription and loss of *Prmt5*-targeting miRNAs. Rather, NF-κB/MYC/mTOR pathways contributed to PRMT5 protein induction via non-transcriptional/non-miRNA mechanisms. Overall, these data are consistent with a model in which initial murine naive T cell activation induces PRMT5 via both transcriptional and non-transcriptional mechanisms downstream of NF-κB, mTOR and/or MYC. During recall activation, memory T cells would be poised to rapidly induce PRMT5 expression, mainly through NF-κB/MYC/mTOR-dependent non-transcriptional mechanisms, possibly allowing the more robust proliferative response characteristic of memory T cell responses. Overall, this study provides insight into the importance of the NF-κB/mTOR/MYC axis in PRMT5-driven pathogenic Th cell responses and may guide targeted therapeutic strategies for MS.

## Materials and Methods

### Mice

B10.PL (Jackson Laboratory) and myelin basic protein (MBP)_Ac1−11_-specific TCR-transgenic (Tg) mice [described previously (46)] were bred in specific pathogen–free conditions at The Ohio State University Laboratory Animal Resources. Murine Pathogen Free (MPF) C57BL/6 and SJL/J mice were purchased from Taconic (Albany, New York). All animal procedures were approved under electronic Institutional Animal Care and Use Committee protocol number 2013A00000151-R1.

### Reagents

Stock Bay11-7082 or Bay11-7085 (SelleckChem), 10058-F4 (Sigma or SelleckChem), LY294002 (SelleckChem), SCH772984 (SelleckChem), and Rapamycin (SelleckChem) were solubilized in DMSO vehicle and diluted ≥1:1,000 for *in vitro* studies.

### Cells

Mouse Th1 and Th2 cell lines were generated from MBP TCR-Tg mice (46) as described previously (10). Th cell lines were not transformed and, therefore, were maintained by stimulation with MBP_Ac1−11_ and irradiated splenocytes in the presence of recombinant human (rh) IL-2 (Miltenyi) every 7–10 days. T cells collected 7–10 days after activation with MBP_Ac1−11_ and irradiated splenocytes provided the resting Th cell condition. To avoid the presence of non-T cells in *in vitro* experiments, resting Th1 or Th2 cell lines were activated with anti-CD3/CD28 for the indicated lengths of time. Mouse naive CD4^+^ T cells were isolated from spleens and lymph nodes using the mouse naive CD4^+^ T cell isolation kit (Miltenyi or STEMCELL Technologies) and activated using 5 μg/ml coated anti-CD3 and 2 μg/ml soluble CD28. Human Th1 and Th2 cells were generated by isolating CD4^+^ T cells with a CD4^+^ T Cell Isolation Kit (STEMCELL Technologies) from human whole blood leukocytes from normal donors and activating on anti-CD3/CD28 Dynabeads (Thermo Fisher) under Th1 (rhIL-12 + anti-hIL-4) or Th2 (rhIL-4 + anti-hIL-12 + anti-hIFNγ) conditions for 1 week and reactivated for further experiments, as previously described (10).

### RNA Isolation and Real-Time PCR

Total RNA was isolated with a mirVana RNA isolation kit (Life Technologies), according to the manufacturer's instructions, and stored at −80°C until analysis. Optical density (OD) 230, 260, and 280 were obtained with a NanoDrop 2000 to evaluate RNA concentration and quality.

For evaluation of mRNA expression, reverse transcription of 100–500 ng RNA was performed using oligo d(T) or random primers and Superscript III (Applied Biosystems) according to manufacturer's instructions; TaqMan quantitative real-time PCR was performed using mouse *Prmt5* (Mm00550472_m1), mouse *Hprt* (Mm0044968_m1), mouse *Myc* (Mm00487804_m1) or human *PRMT*5 (Hs01047356_m1) and human *18S* (Hs99999901_s1) primer sets (Life Technologies), as previously described (47). For miRNA expression, reverse transcription of 2 ng/μl RNA (5 ng/miRNA) was performed using Taqman microRNA reverse transcription kit (Applied Biosystems) and miRNA-specific primers for miR-15a (#000389), miR-15b (#000390), miR-16 (#000391), miR-140-3p (#002234), miR-146a (#000468), miR-146b (#001097), miR-195 (#000494), mouse miR-322/424 (#001076), and human miR-424 (#000604) (Life Technologies), according to manufacturer's instructions. Taqman quantitative real-time PCR was performed using miRNA-specific primers for the above-mentioned miRNAs (Life Technologies), as previously described (47). An initial denaturation step at 95°C for 10 min was followed by 40 cycles of denaturation at 95°C for 15 s and primer annealing/ extension at 60°C for 60 s. Results were analyzed using the comparative Ct method.

### Transfection of Primary Th Cells

Primary naive or memory T cells were transfected with Mirus TransIT-TKO (MIR2150) or TransIT-X2 (MIR6003) transfection reagent according to manufacturer's instructions. Briefly, cells were activated for 4–8 h and subsequently incubated with 25–50 nM of negative control miRNA mimic (4464061 or AM17111), or a combinations of miR-15b (MC10904), miR-140-3p (MC12503), and/or miR-322/424 (MC11080) mimics. Average transfection efficiencies, measured by transfection with Cy3 conjugated negative control miRNA (AM17120), were 30% for naive Th cells, 15–20% for memory Th1 cells and 50% for memory Th2 cells.

### Luciferase Assay

Cos-7 cells were transfected using Lipofectamine (Life Technologies) in serum-free conditions with pmiRGlo Dual Glow Luciferase plasmid (Promega) containing the wild-type mouse *Prmt5* or human *PRMT5* 3′-UTR. In some cases, mouse *Prmt5* 3′UTRs with mutated miRNA-binding sequences were transfected. Mutated 3′UTRs were ordered from IDT. Mouse *Prmt5* 3′UTR was mutated as follows: The miR-140-3p binding site CUGUGGA was mutated to GCCGCCG, the miR-15/16/195/322 binding site UGCUGCU was mutated to CCGGCGC, and the miR-96 sites UGUAGAACAUCUGCUGGUUCAGU and UGCUCAGCCGCCAGA were deleted. Human *PRMT5* 3'UTR was mutated at the putative miR-140-3p binding site CCCUGGA to GCCGCCG. The wild-type or mutant plasmids were co-transfected with miR-15a (MC10235), miR-15b (MC10904), miR-16 (MC10339), miR-96 (MC10422), miR-140-3p (MC12503), miR-195 (MC10827), murine miR-322/424 (MC11080), or human miR-424 (MC10306), or control nonsense (NS) pre-miR (4464061 or AM17111) (Thermofisher). Cells media was changed to complete media (DMEM + 10% Fetal Bovine Serum (FBS) + Penicillin/Streptomycin) after 24 h. Cells were collected and lysed in 1X Cell Lysis Buffer (Promega) 4 days post transfection. Luminol was detected using a GloMax 96 Microplate Luminometer (Promega). Firefly luciferase signal was normalized to Renilla luciferase as a measure of transfection efficiency.

### Western Blotting

Cells were collected at different time points and cell pellets were frozen at −80°C. Cell pellets were lysed in RIPA buffer (10 mM Tris, 150 mM NaCl, 1% Triton X-100, 0.1% SDS, 1% deoxycholate) and protein concentration was quantified by BCA assay. Protein (5–10 μg) was run on 14% SDS-PAGE gels and transferred onto PVDF membrane. Blots were blocked with Odyssey Blocking Buffer (LICOR) and probed with antibodies against PRMT5 (Abcam ab31751), SYM10 pan-symmetric dimethylarginine (Millipore 07–412), or MYC (Cell Signaling #9402). β-actin (Sigma) was used as a housekeeping control. Secondary antibodies used were goat anti-rabbit 800CW and goat anti-mouse 680RD (LICOR). Blots were imaged on an Odyssey CLx machine (LICOR) and quantifications were performed using ImageStudio.

## Results

### TcR-induced NF-κB, mTOR, and MYC Pathways Drive PRMT5 Protein Induction After Naive T Cell Activation

**Figure 1:**
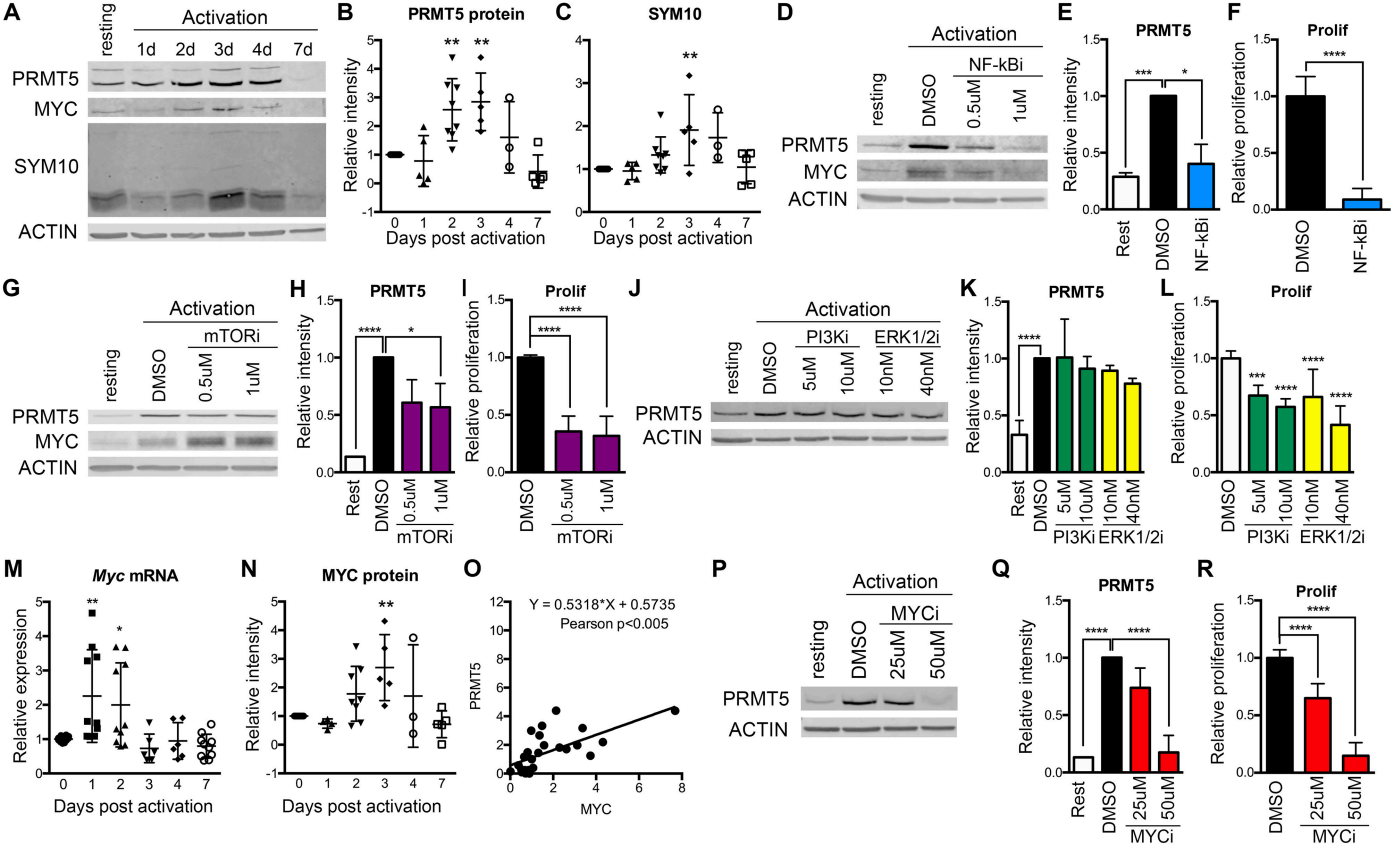
Regulation of PRMT5 protein induction by NF-κB, mTOR and MYC in murine naive Th cells. **(A–C)** PRMT5 and PRMT5′s SYM10 SDM mark expression 0–7 days after murine naive CD4^+^ T cell activation with anti-CD3/CD28. Naive T cells were isolated from 10 to 25 pooled B10.PL mice and analyzed by western blot **(A)** and ImageStudio quantification for PRMT5 **(B)**, SYM10 **(C)**. β-actin was used as a housekeeping control. Data in **(A)** are representative of five independent experiments, which are shown pooled in **(B,C)**. **(D–L)** Isolated murine naive CD4^+^ T cells were treated with the NF-κB inhibitor Bay11-7085 (NF-κBi, **D–F**), the mTOR inhibitor rapamycin (mTORi, **G–I**), the PI3K inhibitor LY294002 (PI3Ki, **J–L**) or the Erk1/2 inihibitor SCH772984 (ERK1/2i, **J–L**). NF-κB, PI3K, or Erk1/2 inhibitors were in culture for the initial 8 h after activation, followed by a drug washout and culture until harvest at 72 h. mTOR inhibitor was present in culture for the entire 72 h period. PRMT5 and MYC protein was analyzed by western blot **(D,G,J)**, quantified using ImageStudio **(E,H,K)**, and T cell proliferation was monitored by ^3^H-thymidine incorporation **(F,I,L)**. **(M–O)** Isolated murine naive CD4^+^ T cells were activated with anti-CD3/CD28 and *Myc* mRNA **(M)** and MYC protein **(N)** were analyzed by Real Time PCR and ImageStudio, respectively, at the indicated time-points. **(O)** Pearson correlation between PRMT5 and MYC protein expression of naive Th cells at day 0–7 after activation. **(P–R)** Isolated naive CD4^+^ T cells were treated with MYC inhibitor 10058-F4 (MYCi) for entire 72 h culture period. PRMT5 protein expression was monitored by western blot **(P)**, quantified using ImageStudio **(Q)**, and proliferation was monitored by ^3^H-thymidine incorporation **(R)**. Data are representative of three independent experiments. ^*^p < 0.05, ^**^p < 0.01, ^***^p < 0.001, ^****^p < 0.0001, one-way ANOVA, followed by Dunnett's multiple comparison test.

We previously reported that PRMT5-selective inhibitors suppressed not only memory Th cell proliferation, but also the proliferation of newly activated naive Th cells, albeit higher concentrations were required (10). This suggested that PRMT5 plays a role during the first encounter of naive T cells with antigen. To determine whether PRMT5 is induced and active after naive Th cell activation, we analyzed PRMT5 expression as well as its symmetric dimethyl arginine mark SYM10 prior to and 1–7 days after anti-CD3/CD28 stimulation (Figures 1A–C). Naive T cells induced PRMT5 protein expression by day 2, similar to what is observed in murine memory T cells (10). PRMT5 induction peaked by day 3 and was subsequently down-regulated from days 4–7, when T cells stop proliferating and return to resting (Figures 1A,B). PRMT5′s SYM10 methylation mark closely followed PRMT5 expression, also peaking 3 days post-activation (Figures 1A,C).

To explore which TcR-induced pathways contribute to PRMT5 induction, we targeted major pathways activated via the TcR, namely NF-κB, PI3K, mTOR, and Erk. Out of these, NF-κB has been shown to promote PRMT5 in cancer cells (21). We found that treatment with NF-κB inhibitor Bay11-7085 for the initial 8 h after naive Th cell activation strongly suppressed PRMT5 protein induction (Figures 1D,E, 60% decrease) and proliferation (Figure 1F) at 3 days post activation. The mTOR pathway inhibitor rapamycin also suppressed PRMT5 protein induction (Figures 1G,H, 43% decrease) and proliferation (Figure 1I), albeit to a lower extent. In contrast, 8 h treatment with the PI3K inhibitor LY294002 or the MAPK/Erk inhibitor SCH772984 had minimal effects on PRMT5 induction (Figures 1J,K), albeit still having some impact on proliferation through PRMT5-independent pathways (Figure 1L). These data indicate that the NF-κB and mTOR pathways are major drivers of PRMT5 protein induction in T cells.

Another potential regulator of PRMT5 is MYC, which has been reported to transcriptionally promote PRMT5 expression in transformed cells (22, 23). Interestingly, MYC induction has been reported downstream of both NF-κB and mTOR pathways in transformed cells and myeloid progenitors (48–50). Consistent with MYC driving PRMT5 in T cells, we found that both *Myc* transcript (Figure 1M) and MYC protein (Figures 1A,N) were induced after T cell activation, mirroring PRMT5′s protein expression pattern (Figure 1O). MYC induction after T cell activation required NF-κB (Figure 1D, 0.61 ± 0.14 relative to DMSO) but not mTOR (Figure 1G, 1.92 ± 0.49 relative to DMSO) activity. To determine the importance of MYC signaling during T cell activation, we treated naive T cells with MYC inhibitor 10058-F4 and found suppressed PRMT5 protein induction (Figures 1P,Q) and T cell proliferation (Figure 1R). These data are consistent with a scenario in which MYC mediates PRMT5 induction downstream of NF-κB.

### Transcriptional and Non-transcriptional Mechanisms for PRMT5 Modulation Are Active After Initial Murine Naive CD4^+^ T Cell Activation

**Figure 2:**
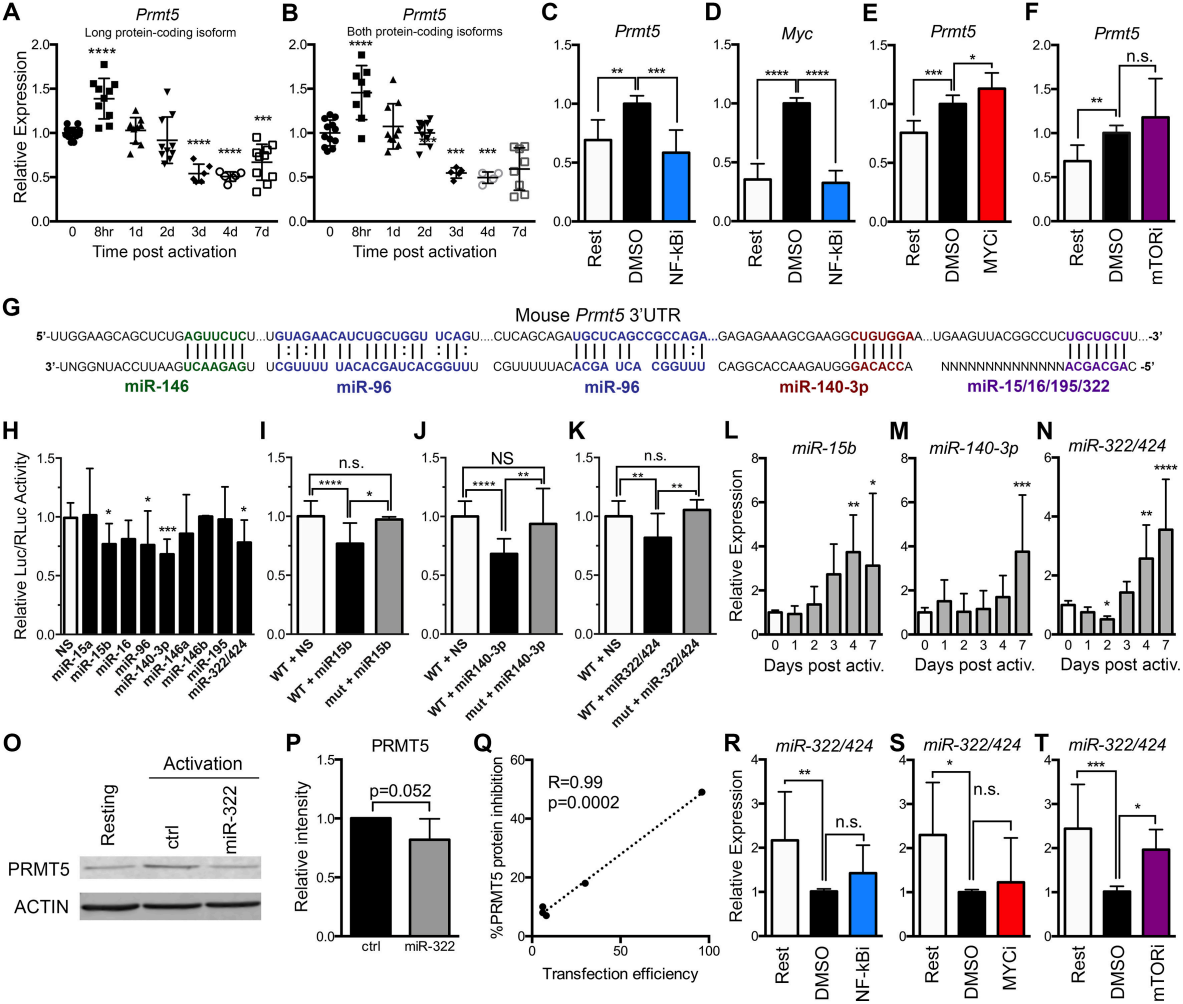
PRMT5 protein induction is regulated transcriptionally and post-transcriptionally in murine naive Th cells. **(A,B)** Murine naive Th cells were isolated from B10.PL mice, activated with anti-CD3/CD28 and analyzed by real time PCR for *Prmt5* transcripts (exon 12–13) that yield the long PRMT5 protein isoform **(A)** or transcripts (exon 7–8) that can yield both the long or short PRMT5 protein isoform **(B)** at the indicated time-points. **(C–E)** Isolated naive Th cells were activated for 8 h and treated with NF-κB inhibitor Bay11-7082 (NF-κBi) **(C–D)**, MYC inhibitor 10058-F4 (MYCi) **(E)**, or mTOR inhibitor rapamycin **(F)**, and *Prmt5* **(C,E,F)** or *Myc* **(D)** mRNA were measured by real time PCR. **(G)** Mouse *Prmt5* 3′UTR and Targetscan or RNA22-predicted miRNA-binding sites. The miRNA sequence is shown below the *Prmt5* mRNA sequence (straight lines indicate a Watson-Crick base pairing between mRNA and miRNA nucleotides and dotted lines indicate wobble base pairing). Bolded nucleotides indicate the seed sequence of the miRNAs. **(H)** Cos-7 cells were transfected with pmiRGlo Dual Glo Luciferase plasmid containing mouse *Prmt5* 3′UTR and transfected with nonsense (NS) miRNA, miR-15a, miR-15b, miR-16, miR-140-3-p, miR-146a, miR-146b, miR-195, or miR-322/424. Firefly luciferase expression was normalized to Renilla luciferase expression. **(I–K)** Cos-7 cells were transfected with pmiRGlo Dual Glo Luciferase plasmid containing mouse wild-type or mutated *Prmt5* 3′UTR and the indicated miRNAs: miR-15b **(I)**, miR-140-3p **(J)**, or miR-322/424 **(K)**. The binding sites mutated in plasmids used in **(I–K)** are indicated in Materials and Methods section. For **(H–K)**, data are pooled from 3 independent experiments per miRNA tested. One-way ANOVA, followed by Dunnett's multiple correction test. **(L)** *miR-15b*, **(M)** *miR-140-3p* and **(N)** *miR-322/424* expression was analyzed by real time PCR. Data are pooled from five independent experiments (*n* = 10). **(O–Q)** Isolated naive T cells were activated and transfected with a control or miR-322/424 miRNA mimic and PRMT5 and ACTIN protein expression was analyzed by Western blot **(O)**, and quantified by ImageStudio **(P)**, *t*-test *p* = 0.052, in five independent experiments. **(Q)** Pearson correlation analysis between the levels of transfection efficiency, measured by a Cy3-conjugated miRNA mimic, and level of PRMT5 suppression. **(R–T)** Isolated naive T cells were activated for 24 h, treated with NF-κBi **(R)**, MYCi **(S)**, or mTORi **(T)**, and miR-322/424 expression was analyzed by real time PCR. One-way ANOVA, followed by Dunnett's multiple correction test. ^*^p < 0.05, ^**^p < 0.01, ^***^p < 0.001, ^****^p < 0.0001.

MYC-driven *Prmt5* transcription has been shown to mediate PRMT5 induction in transformed cells (23) and may similarly drive PRMT5 induction in T cells. To address this question, we evaluated *Prmt5* expression in murine resting and activated naive Th cells. *Prmt5* mRNA underwent transient induction at 8 h, followed by a decrease 3–7 days post–naive T cell activation (Figures 2A,B). These effects were observable in assays that recognize either both protein-coding isoforms of *Prmt5* (Figure 2B) or the long protein-coding isoform (Figure 2A). Overall, these results suggest that a burst of enhanced *Prmt5* transcription after T cell activation contributes to transient PRMT5 protein induction in recently activated naive T cells.

To then evaluate which signaling pathways are behind transcriptionally-mediated induction of PRMT5 protein, we treated naive T cells with MYC, mTOR and NF-κB inhibitors and evaluated their impact on *Prmt5* transcripts. We found that treatment with NF-κB inhibitor Bay11 suppressed *Prmt5* mRNA induction (Figure 2C). Since NF-κB has been reported to promote *Myc* transcription in cancer cells (22, 23), we hypothesized that NF-κB-driven MYC expression promoted *Prmt5* transcription. Initially supporting this model, NF-κB signaling was required for *Myc* transcription (Figure 2D) and MYC protein expression (Figure 1D) in T cells. However, even though MYC inhibitor 10058-F4 substantially suppresses PRMT5 protein expression (Figures 1P,Q), it did not suppress, but rather enhanced, *Prmt5* mRNA induction (Figure 2E). Further, mTOR inhibitor rapamycin had no effect on *Prmt5* mRNA induction (Figure 2F). The latter data indicate that in naive T cells, contrary to what was known from transformed cells (22, 23), NF-κB drives *Prmt5* transcription while mTOR and MYC drive PRMT5 protein induction via non-transcriptional mechanisms.

Beyond transcription, gene expression can be regulated at the post-transcriptional level, such as mRNA processing or miRNA control, and the translational level, influencing the initiation or termination of protein translation from mRNA. Regulation of PRMT5 via miR-96/92b has been reported in cancer cells, in which loss of these miRNAs enhances the pool of mRNA available for PRMT5 translation (19, 21). We hypothesized that loss of *Prmt5*-targeting miRNAs after activation may contribute to PRMT5 protein induction. miR-96 was predicted to target *Prmt5* transcripts by RNA22, a tool that computes miRNA target predictions (Figure 2G). In addition, miR-15a, miR-15b, miR-16, miR-140-3p, miR-146a, miR-146b miR-195, and miR-322/424 were predicted by TargetScan algorithms to target the 3′-untranslated region (UTR) of murine *Prmt5* (Figure 2G). To validate that these miRNAs indeed bind *Prmt5*'s 3′-UTR and directly suppress protein expression, we performed luciferase assays using a plasmid carrying the firefly luciferase gene and the *Prmt5* 3′-UTR. miRNAs that target *Prmt5* are expected to suppress luciferase expression, and miR-15b, miR-140-3p and miR-322/424 suppressed luciferase activity between 20 and 30% (Figure 2H). miR-96 also suppressed luciferase activity (Figure 2H) but this effect was unrelated to the *Prmt5* 3′UTR, as miR-96 had the same effect in the luciferase vector that did not carry the *Prmt5* 3′UTR (Supplemental Figure 1A). In contrast, miR-15a, miR-16, miR-146a/b, and miR-195 did not suppress luciferase activity (Figure 2H), indicating these miRNAs do not target *Prmt5*. Next, the miRNA-binding sites for validated miRNAs were mutated to confirm specific miRNA binding to the *Prmt5* 3′UTR. When the miRNA binding sites for miR-15b, miR-140-3p and miR-322/424 were mutated, luciferase activity was recovered (Figures 2I–K), indicating that these miRNAs directly and specifically suppress PRMT5 protein expression.

To test the biological relevance of these miRNAs, we measured their expression after naive Th cell activation. If these miRNAs contribute to PRMT5 protein expression after naive T cell activation, they would be expected to decrease after activation, to allow PRMT5 translation. Contrary to this possibility, we found that the majority of the tested miRNAs were relatively stable from 0 to 2 days after activation and then progressively increased, reaching peak expression 4 to 7 days post activation (Figures 2L–N, Supplemental Figures 1B–G). Interestingly, the induction and highest expression of *Prmt5*-targeting miRNAs (Figures 2L–N) coincided with the down-regulation of PRMT5 protein observed from days 4 to 7, as T cells return to a resting condition (Figure 1A). Only one miRNA, miR-322/424 was significantly downregulated 2 days post-activation (Figure 2N), a decrease that could contribute to translation of *Prmt5* transcripts to protein. Although primary naive T cells are notoriously difficult to transfect, we attempted to overexpress miR-322/424 via transfection. We only observed a trend decrease in PRMT5 protein expression when all experiments of variable transfection efficiencies were combined (Figures 2O,P). Nonetheless, correlation analyses showed a clear positive correlation between transfection efficiency and PRMT5 protein repression, reaching a 50% decrease with 96% transfection efficiency (Figure 2Q). We next tested the contribution of NF-κB, MYC or mTOR pathways to miR-322 expression down-regulation and PRMT5 protein induction. While NF-κB and MYC inhibitor treatment had no effect on miR-322 expression (Figures 2R,S), blocking mTOR signaling with rapamycin restored miR-322 expression to levels close to those observed in resting naive T cells (Figure 2T). These results are consistent with mTOR-dependent miR-322/424 loss contributing to initial PRMT5 induction in activated naive T cells.

Together, these data support a model in which both NF-κB-dependent transcriptional induction of *Prmt5* and mTOR and MYC-dependent non-transcriptional mechanisms mediate PRMT5 protein induction in activated naive T cells. mTOR was required for miR-322/424 suppression, and therefore likely induces PRMT5 by relieving miRNA-mediated suppression of PRMT5 translation. In contrast, NF-κB-dependent MYC induction promotes PRMT5 protein via a non-transcriptional/non-miRNA-dependent mechanism. While the exact mechanism is beyond the scope of this manuscript, MYC has been shown to enhance translation via transcription-independent mechanisms (51). While miRNAs may play a minor role during initial PRMT5 induction in naive T cells, it is conceivable that high miR-15b, miR-140-3p and miR-322/424 induction at days 4–7 after T cell activation may contribute to PRMT5 protein down-regulation in non-proliferative, resting memory T cells. If that is the case, one would expect suppression of these miRNAs after recall memory T cell activation to allow PRMT5 induction and T cell proliferation.

### Transcriptional and Non-transcriptional Regulation of PRMT5 in Murine Memory Th Cells

**Figure 3:**
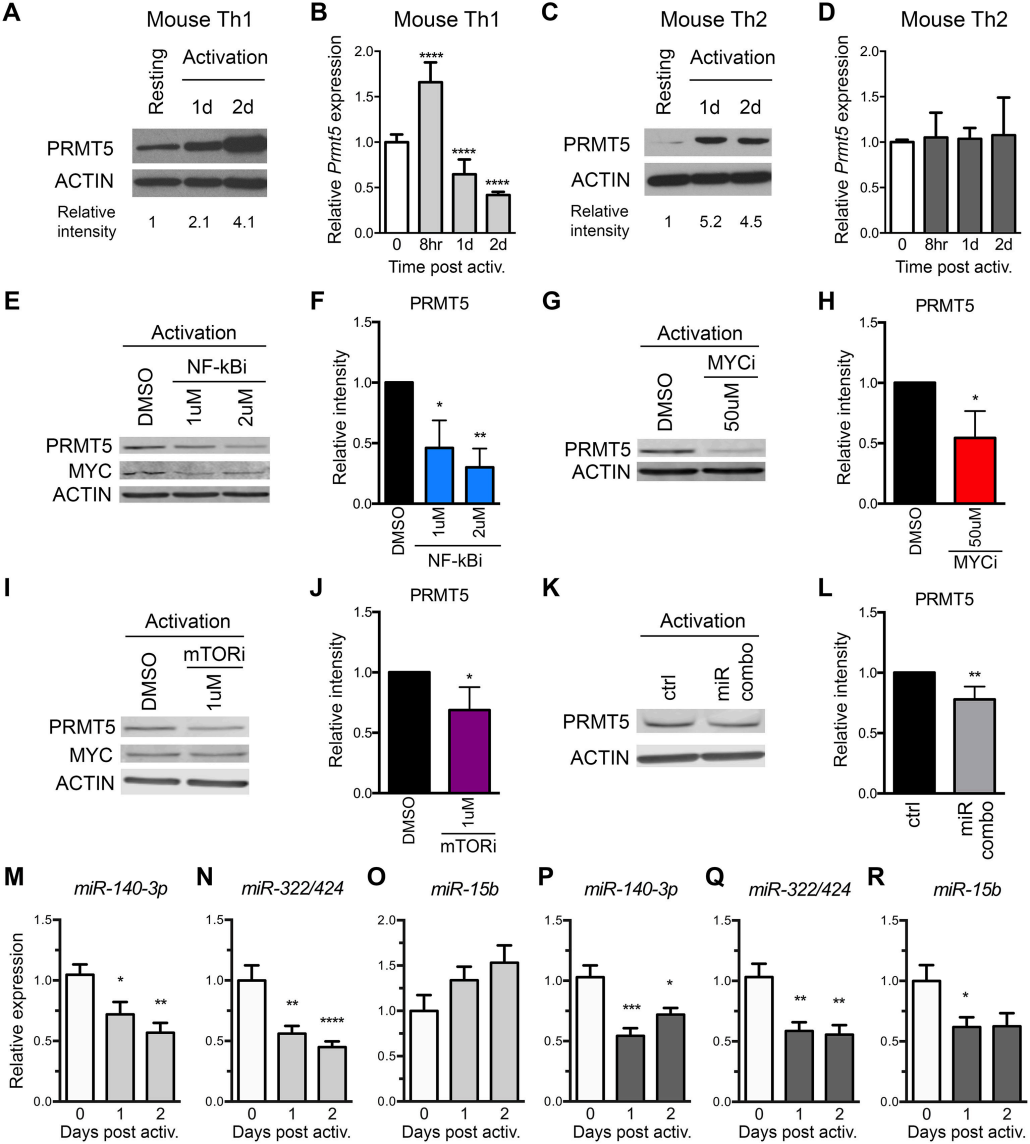
*Prmt5*-targeting miRNAs are downregulated after memory murine Th cell activation. **(A–D)** Murine memory MBP TcR transgenic Th1 **(A,B)** or Th2 **(C,D)** line cells (10) were activated with anti-CD3/CD28 and PRMT5 protein expression (**A**,**C**, western blot) and *Prmt5* mRNA transcripts (**B,D**, real-time PCR) were analyzed at the indicated time-points. Resting cells are Th1/Th2 line cells 7 days after restimulation with antigen presenting cells and MBP. Data are pooled from 3 to 4 independent experiments. **(E–J)** Memory mTh1 cells were activated and treated with NF-κB inhibitor Bay11 (NF-κBi), MYC inhibitor 10058-F4 (MYCi), mTOR inhibitor rapamycin (mTORi), or DMSO vehicle control. PRMT5 and MYC protein expression were measured by Western blot **(E,G,I)** and quantified by ImageStudio **(F,H,J)**. ACTIN is used as a housekeeping control. **(K,L)** Memory Th cells were activated and transfected with a combination of miR-15b, miR-140-3p and miR322/424. PRMT5 protein expression was analyzed by western blot **t** and quantified by ImageStudio **(L)**, *n* = 5. Data are pooled from three independent experiments. Experiments in which transfection efficiency was lower than 20% were excluded from analysis. *miR-140-3p*, **(M,P)** *miR-322/424* **(N,Q)**, and *miR-15b* **(O,R)** expression was analyzed by Real Time PCR in Th1 (**M–O**, light gray bars) and Th2 (**P–R**, dark gray bars) and expressed as fold change relative to resting baseline. Data are pooled from four independent experiments (*n* = 8). ^*^p < 0.05, ^**^p < 0.01, ^***^p < 0.001, ^****^p < 0.0001, one-way ANOVA, followed by Dunnett's multiple correction test.

To address the role of both transcriptional and miRNA-mediated post-transcriptional mechanisms of PRMT5 induction in recall T cell responses, we used MBP-specific TcR transgenic memory Th1 and Th2 cell lines. These cells are kept via weekly MBP/irradiated feeder cell stimulation, which induces PRMT5 expression and proliferation (10). Seven days from prior antigenic stimulation, cells return to a resting state, characterized by low PRMT5 expression and low proliferation (10). As previously reported (10), PRMT5 protein expression was induced after T cell activation in murine MBP TcR Tg memory Th1 (Figure 3A) and Th2 cells (Figure 3C). Since PRMT5 was induced transcriptionally in naive T cells, we asked whether PRMT5 is induced transcriptionally in memory murine Th cells. Similar to what we observed in naive Th cells, *Prmt5* mRNA expression was transiently induced at 8 h, followed by a decrease 1–2 days post T cell activation in murine Th1 cells (Figure 3B) but not in Th2 cells (Figure 3D). To determine whether similar TcR-induced signaling pathways regulate PRMT5 expression in naive vs. memory T cells, we treated Th1 cells with NF-κB and MYC inhibitors. Similar to observations in naive T cells, PRMT5 protein induction was dependent on NF-κB (Figures 3E,F), MYC (Figures 3G,H), and mTOR (Figures 3I,J) signaling. Further, MYC protein induction was dependent on NF-κB (Figure 3E, 0.7 ± 0.01 relative to DMSO), but not mTOR (Figure 3I, 1.2 ± 0.2, relative to DMSO) activity. However, the mechanisms by which PRMT5 expression is regulated by these pathways differed. Although NF-κB and MYC inhibitor, but not mTOR inhibitor, treatment suppressed *Myc* transcription (Supplemental Figures 2B,D,F), none of these pathways were required for *Prmt5* transcriptional induction (Supplemental Figures 2A,C,E). These data point to NF-κB, mTOR, and MYC pathways driving PRMT5 protein induction in memory Th1 cells via non-transcriptional mechanisms, which may include miRNA suppression or translation enhancement.

The observation that several *Prmt5*-targeting miRNAs are induced as cells become resting memory led us to hypothesize that miR-15b, miR-140-3p, and miR-322/424 would play a larger role in regulating PRMT5 expression after activation of memory T cells. To test this hypothesis, we transfected memory Th cells with the combination of *Prmt5*-targeting miRNAs, miR-15b, miR-140-3p, and miR-322/424. With transfection efficiency ranging from 20 to 50%, overexpression of *Prmt5*-targeting miRNAs resulted in a small but consistent (15–30%) reduction in PRMT5 protein expression in activated memory Th cells (Figures 3K,L). Further, we analyzed expression of validated *Prmt5*-targeting miRNAs miR-15b, miR-140-3p and miR-322/424, expecting decreased expression after T cell activation (Figures 3M–R). Only miR-140-3p and miR-322/424 were downregulated after memory T cell activation in both Th1 (Figures 3M,N) and Th2 cells (Figures 3P,Q). While miR-15b was not down-regulated in Th1 cells (Figure 3O), it decreased in Th2 cells (Figure 3R). Other miRNAs initially predicted to target *Prmt5* but not validated by luciferase assay were modulated to various extents after Th1 (Supplemental Figures 2G–L) or Th2 cell activation (Supplemental Figures 2M–R). Unexpectedly, we found that *Prmt5*-targeting miRNAs miR-15b and miR-140-3p were not regulated by NF-κB (Supplemental Figures 2S,T), MYC (Supplemental Figures 2U,V) or mTOR (Supplemental Figures 2W,X) pathways in memory Th1 cells. Overall, these results are consistent with a major role for non-transcriptional NF-κB, mTOR and MYC-mediated PRMT5 induction in murine memory Th1 cells.

### PRMT5 Protein Induction Is Regulated Transcriptionally and Post-transcriptionally in Human Memory T Cells

**Figure 4:**
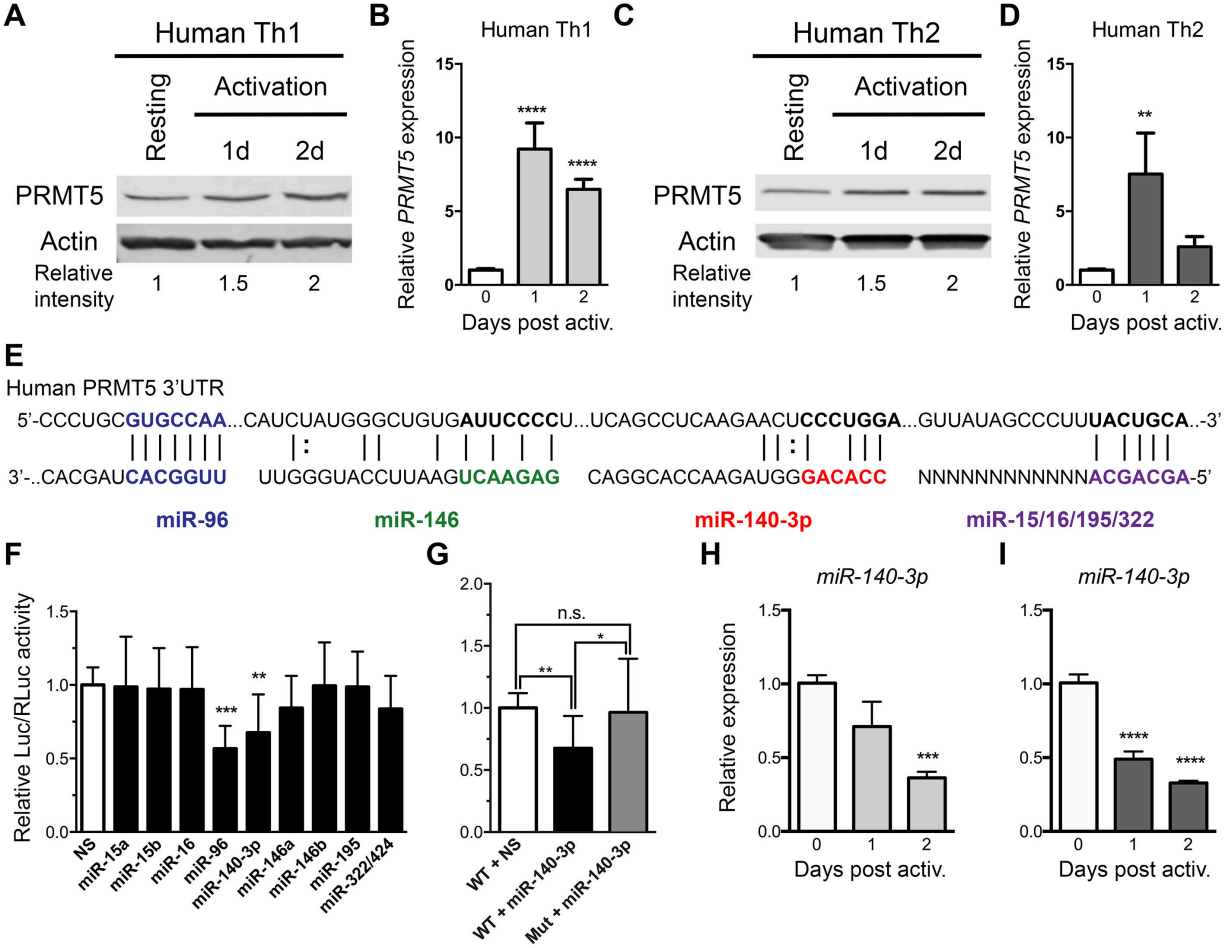
PRMT5-targeted miRNAs are downregulated after memory human Th cell activation. **(A–D)** Human memory Th1 **(A,B)** or Th2 **(C,D)** cells were activated with anti-CD3/CD28 and PRMT5 protein expression (**A,C**, western blot) and *PRMT5* mRNA transcripts (**B,D**, Real-Time PCR) were analyzed at the indicated timepoints. Resting cells correspond to Th1/Th2 cells (after two rounds of differentiation) and 7 days after last stimulation. **(E)** Human *PRMT5* 3′UTR and predicted miRNA-binding sites. The miRNA sequence is shown below the *PRMT5* mRNA sequence (straight lines and dotted lines connecting the mRNA and miRNA nucleotides indicate Watson-Crick base pairing and wobble base pairing, respectively). Bolded nucleotides indicate the seed sequence. **(F)** Cos-7 cells were transfected with pmiRGlo Dual Glo Luciferase plasmid containing human *PRMT5* 3′UTR and miR-15a, miR-15b, miR-96, miR-16, miR-140-3p, miR-146a, miR-146b miR-195, miR-322/424 **(G)** Cos-7 cells were transfected with pmiRGlo Dual Glo Luciferase plasmid containing human WT or mutated *PRMT5* 3′UTR and miR-140-3p. Firefly luciferase expression was normalized to Renilla luciferase expression and expressed as a ratio of Luc/RLuc relative to nonsense (NS) control. **(H,I)** miR-140-3p expression was analyzed by real time PCR in Th1 **(H)** and Th2 **(I)** cells after T cell activation. Data are representative of three independent experiments. Error bars represent *SD*. One-way ANOVA, followed by Dunnett's multiple comparison test. ^*^p < 0.05, ^**^p < 0.01, ^***^p < 0.001, ^****^p < 0.0001.

As previously reported (10) and similar to mouse memory Th cells, PRMT5 protein expression is induced after human Th1 (Figure 4A) and Th2 (Figure 4C) cell activation. We explored whether PRMT5 protein was induced transcriptionally after recall activation of human memory T cells. In human Th1 and Th2 cells, *PRMT5* transcripts were induced in a more robust and prolonged manner (Figures 4B,D) than that observed in equivalent murine memory T cells (Figures 3B,D), maintaining high expression up to 2 days after activation.

We next explored whether PRMT5 protein induction was similarly regulated at the transcriptional and/or post-transcriptional level in human Th cells. miR-96 has been shown to suppress PRMT5 translation in human B cell lymphoma cells and human and mouse *PRMT5* 3′UTRs are highly conserved at the predicted target regions of our miRNAs (Figure 4E), suggesting that miR-15a, miR-15b, miR-16, miR-140-3p, miR-322/424, and others could potentially regulate human PRMT5. To test this, we generated a luciferase plasmid carrying the human *PRMT5* 3′UTR and tested the impact of various human miRNAs on luciferase activity. Out of the predicted *PRMT5*-targeting miRNAs, only miR-140-3p significantly suppressed luciferase activity, approximately 32% (Figure 4F). miR-96 also suppressed luciferase activity (Figure 4F), though non-specifically, as this miRNA suppressed luciferase activity in the presence of a plasmid that did not contain the *PRMT5* 3′UTR (Supplemental Figure 1A). Mutation of the miR-140-3p binding site restored luciferase activity to control levels (Figure 4G), confirming that this miRNA specifically targets the human *PRMT5* 3′UTR. To determine whether this miRNA indeed decreases after recall T cell activation, we evaluated its expression by Real-Time PCR in human memory Th1 and Th2 cells. miR-140-3p was downregulated after Th1 Figure 4H and Th2 cell Figure 4I activation, suggesting that loss of this miRNA could contribute to PRMT5 protein upregulation in human Th cells. Although not validated to target PRMT5, several other tested miRNAs were differentially modulated after human Th1 (Supplemental Figures 3A–F) and Th2 cell activation (Supplemental Figures 3G–L). Several miRNAs were significantly reduced after Th1 (Supplemental Figures 3B–D) and Th2 (Supplemental Figures 3H–J) cell activation, including miR-15a, miR-15b, and miR-195. Overall, these data indicate that, although transcriptional regulation is likely the most prominent mechanism for PRMT5 induction in human memory T cells, PRMT5 protein expression can be regulated by both transcriptional and post-transcriptional mechanisms in human T cells.

## Discussion

The arginine methyltransferase PRMT5 is induced during recall memory Th cell responses, promoting T cell expansion, inflammatory responses and EAE. Here, we show that PRMT5 protein and activity is similarly induced during initial activation of naive T cells and that PRMT5 protein induction is largely dependent on NF-κB, mTOR and MYC in both naive and memory T cells. However, the exact underlying mechanisms differ. While NF-κB, mTOR and/or MYC contributed to both transcriptional and non-transcriptional induction of PRMT5 in naive T cells, these pathways only mediated non-transcriptional induction in memory T cells. Loss of *Prmt5*-targeting miRNAs was also observed in activated T cells, particularly memory T cells, suggesting they further contribute to PRMT5 regulation. Transcriptional and post-transcriptional mechanisms for PRMT5 induction were also observed in human memory T cells. Overall, our data show that PRMT5 protein induction is conserved from naive to memory and from mouse to human T cell responses. This process appears to be regulated by dynamic mechanisms that include transcriptional and non-transcriptional induction and miRNA-mediated post-transcriptional repression.

Naive and memory Th cells have distinct requirements and different kinetics for activation following antigenic stimulation. Thus, it is not surprising that, while NF-κB/MYC/mTOR pathways contribute to induction of PRMT5 in both types of T cells, the underlying mechanisms differ. Naive Th cells have a higher signaling threshold for T cell activation, requiring longer and stronger TcR/co-stimulatory signals in order to induce proliferation (52, 53). Memory Th cells are in contrast less dependent on costimulation and can rapidly proliferate and perform effector functions after antigen encounter (52, 53). Our data shows that several TcR-induced signaling pathways, including NF-κB, mTOR, and MYC, are required to promote PRMT5 protein expression in naive Th cells. However, PI3K and Erk pathways are largely dispensable for PRMT5 expression. Classically, mTOR pathway activation occurs downstream of PI3K. Thus, it is surprising that mTOR inhibition suppressed PRMT5 protein expression, though PI3K inhibition did not. In support of this data, mTOR activation has also been shown to occur independently of PI3K activation in CD8^+^ T cells (54). Loss of *Prmt5*-targeting miRNA miR-322/424 at 48 h may further promote PRMT5 protein expression in naive Th cells. These data support that there are high requirements to drive initial PRMT5 protein upregulation, and subsequent proliferation in naive Th cells. Subsequent increase of *Prmt5*-targeting miRNAs between days 4 and 7 after activation may then contribute to downregulation of PRMT5 protein expression, reaching its lowest levels at day 7, when the Th cells have reached a resting, experienced, or memory, state. Restimulation of memory Th cells results in rapid downregulation of *Prmt5*-targeting miRNAs, miR-15b, miR-140-3p, and miR-322/424 and rapid NF-κB/MYC/mTOR-dependent upregulation of PRMT5 protein expression, suggesting that memory Th cells are poised to upregulate PRMT5 protein expression more quickly after activation, allowing the faster proliferative and effector function characteristic of memory cells.

**Figure 5:**
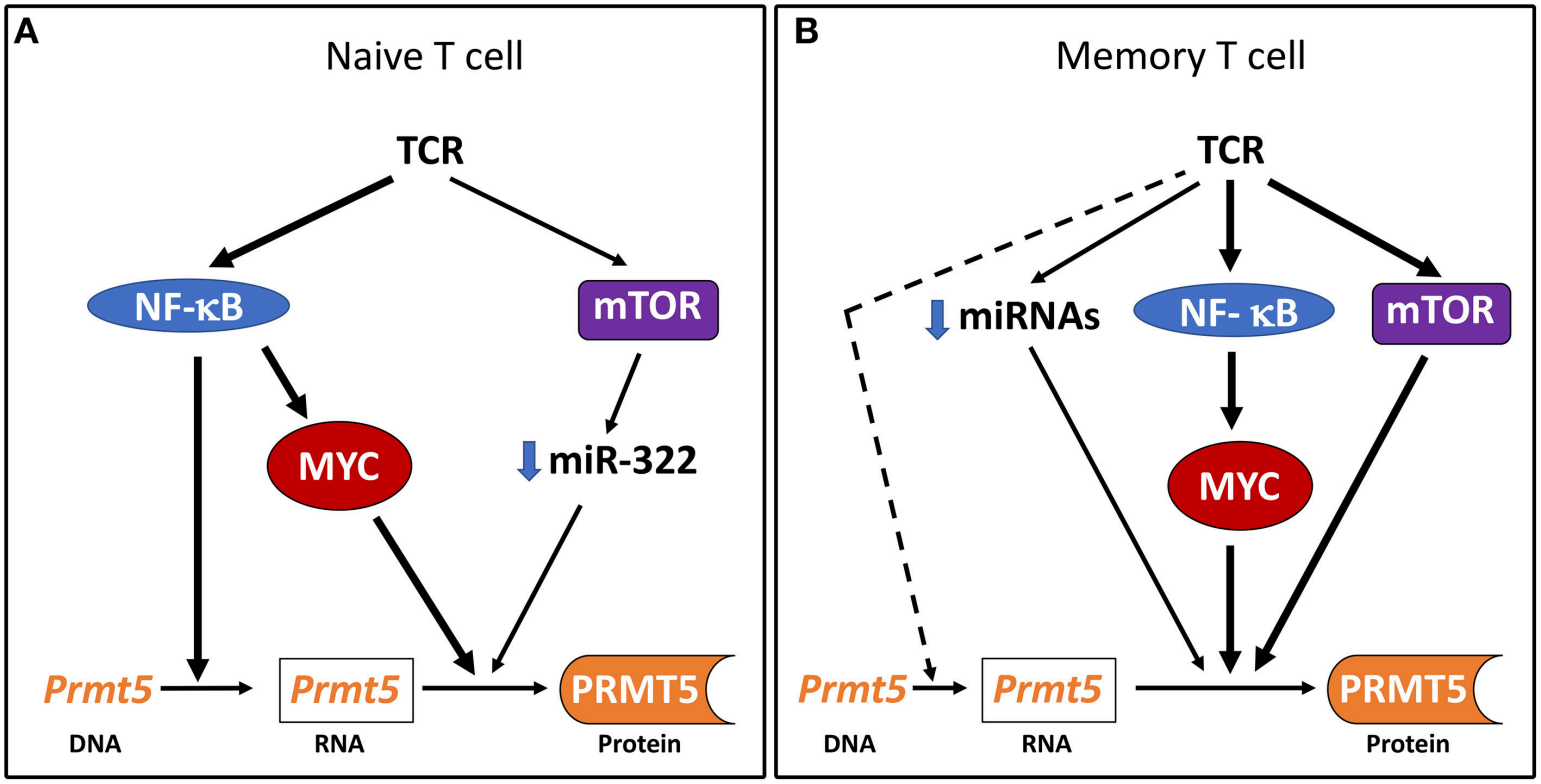
Model of PRMT5 expression regulation after Th cell activation. **(A)** In naive T cells, NF-κB drives *Prmt5* transcription and the NF-κB/mTOR/MYC axis drives PRMT5 protein induction through post-transcriptional and/or translational mechanisms. **(B)** In memory T cells, the NF-κB/mTOR/MYC axis drives PRMT5 protein induction through predominantly non-transcriptional mechanisms. *Prmt5* transcription and miRNA loss may further contribute to PRMT5 induction via NF-κB/mTOR/MYC-independent mechanisms.

This study identifies the NF-κB/mTOR/MYC axis as an important driver of PRMT5 protein expression in Th cells. Both NF-κB and mTOR, as well as downstream target MYC, are required for full PRMT5 protein induction and NF-κB is required for PRMT5 transcription in naive Th cells (Figure 5A). Based on previous transformed cells studies (21–23), our initial hypothesis was that NF-κB would drive PRMT5 protein expression via miRNA loss and MYC-dependent *Prmt5* transcription. Instead, we found that NF-κB did not regulate *Prmt5*-targeting miRNAs and MYC activity was not required for *Prmt5* transcription, suggesting NF-κB can independently drive *Prmt5* transcription in naive T cells. Further, in contrast to data found in cancer cells (49), mTOR activity was dispensable for MYC protein induction in naive and memory murine T cells, regulating PRMT5 expression via downregulation of *Prmt5*-targeting miRNAs or alternate mechanisms (Figures 5A,B). These data support that PRMT5 expression is regulated differently in normal T cells compared to transformed cancer cells. Further analyses of these differences may provide an opportunity to selectively target cancer cell proliferation and reduce the characteristic immunosuppressive effects of chemotherapy. Nonetheless, NF-κB still promotes *Myc* transcription and protein expression, consistent with transformed cell data (48), and MYC activity was required for PRMT5 protein induction. While our and previous studies show that MYC drives PRMT5 (22, 23), PRMT5 in turn drives MYC expression (55, 56), suggesting that a MYC-PRMT5-MYC positive feedback loop could further enhance PRMT5 expression in activated Th cells.

A major role for non-transcriptional mechanism for MYC-driven PRMT5 protein induction, particularly in memory T cells, is a novel and unexpected finding. We did not find evidence that MYC induces PRMT5 via miRNAs and mTOR was only required for miR-322 loss in naive T cells. Alternative mechanisms include post-transcriptional regulation, via modulation of RNA processing, stability or miRNAs, or translational regulation, via modulation of translation initiation or termination. Although MYC has primarily been studied as a transcriptional regulator, MYC can also regulate protein translation via multiple mechanisms, including eukaryotic initiation factor (eIF) 4-mediated translation initiation (23, 57, 58) and mRNA cap methylation(51, 59). T cell activation is followed by large changes in gene expression, a portion of which corresponds to eIF4-mediated translational enhancement (60). The fact that eIF4, and presumably its translational effects, are required for T cell proliferation (60) suggests the possibility that eIF4 is required for PRMT5 expression. mTORC1 signaling promotes eIF4e-mediated translational regulation (61). Since we don't observe sustained induction of *Prmt5* mRNA in mouse naive or memory Th cells, our data point to a need for increased translational stability to sustain PRMT5 protein expression during T cell activation. Interestingly, MYC also selectively promotes mRNA cap methylation and subsequent translation of a subset of mRNAs, supporting that this is a possible mechanism by which MYC regulates PRMT5 protein induction during T cell activation. Additionally, PRMT5 may utilize similar pathways for its downstream function. This possibility is supported by the finding that PRMT5-mediated SDM regulates eIF4e-mediated 5'-cap-dependent (62) and IRES-dependent translation (55). While these possibilities are intriguing, additional studies will be required to address the role of translation on PRMT5 induction and/or function in T cells.

Post-transcriptional miRNA-mediated regulation of PRMT5 expression has long been known to operate in transformed cells (19, 21) and was therefore our initial favored hypothesis for PRMT5 regulation in T cells. We found and validated several *Prmt5/PRMT5*-targeting miRNAs in mouse and human T cells. In the early phases after naive T cell activation, only miR-322/424 decreased significantly, suggesting that most of PRMT5 induction occurs via the transcriptional and non-transcriptional mechanisms described in the paragraph above. However, miR-322/424, miR-15b, and miR-140-3p substantially increase as naive T cells down-regulate PRMT5 and return to the resting non-proliferative state of resting memory cells, suggesting these miRNAs contribute to PRMT5 suppression. Once these resting memory T cells were reactivated, *Prmt5-*targeting miRNAs decreased, which may contribute to PRMT5 protein induction and fast proliferative responses in recall memory T cell responses. Overexpression of *Prmt5*-targeting miRNAs in our system resulted in modest decreases in PRMT5 protein. miRNAs suppressing PRMT5 protein expression would be expected to suppress T cell proliferation as well. One limitation is that, since efficient transfection of primary Th cells is difficult to achieve, a majority of untransfected cells continue to proliferate, overwhelming the culture and making it difficult to observe an effect. Thus, it is conceivable that miRNAs may have a larger effect on PRMT5 protein expression than our data may initially suggest.

We have previously shown that PRMT5 drives inflammatory Th cell expansion and EAE. However, a direct link between PRMT5 and human MS has yet to be proven. It is nonetheless of interest that NF-κB and its downstream signaling have been strongly linked to MS. MS-associated risk alleles have been identified in more than 100 NF-κB pathway genes (43, 63). In addition, NF-κB signaling is overactive in MS patients and functional studies have shown that NF-κB pathway SNPs rs228614, rs228614 and rs1800693 can alter NF-κB component expression and lead to increased NF-κB activity and inflammatory cytokine production (44). We had previously shown that NF-κB activity is required for PRMT5 induction in human memory Th1 cells (10) and now provide an additional link between NF-κB activity and PRMT5 expression in naive and memory mouse Th cells (Figures 1, 3). This suggests that increased NF-κB activity in MS patients may act as a PRMT5 driver, contributing to overactive inflammatory T cell responses in MS. Recently, an MS risk-associated SNP has been identified in *MYC*. Although the implications of this SNP on MYC activity and PRMT5 expression remain to be fully elucidated, we show here that MYC activity is required for full PRMT5 induction in T cells. Finally, miRNAs have also been shown to be dysregulated in MS patients (47, 64). In particular, *Prmt5-*targeting miRNA miR-15b is downregulated in MS whole blood (65) and serum (66). Low levels of miR-15b has been linked to increased mTOR signaling (67), increased Th17 differentiation (68), and decreased Tregs (67). Thus, it is possible that loss of *PRMT5*-targeting miRNAs could contribute to increased PRMT5 protein expression and MS.

Altogether, our study sheds light on the pathways and mechanisms driving PRMT5 protein expression in naive and memory Th cells. Because PRMT5 appears to be important in driving autoimmune T cell responses (10), our data support the therapeutic potential of modulating PRMT5 expression by targeting NF-κB, mTOR or MYC pathways. Further understanding of upstream PRMT5 regulators and downstream PRMT5 targets in activated Th cells should provide novel and useful therapeutic targets in the treatment of T cell-mediated autoimmune diseases such as MS.

## Ethics Statement

All animal studies were performed in accordance to the guidelines of the Animal Welfare Act (AWA) and the Policy on Humane Care and Use of Laboratory Animals (PHS). Animal use protocol was approved by the Institutional Animal Care and Use Committee (IACUC). Studies using human T cells were all completely de-identified and therefore, exempt from Institutional Review Board (IRB) approval.

## Author Contributions

LW, JN, SA, and MG-d-A formulated research hypothesis and designed experiments. LW, JN, SA, SS, and GN performed experiments and interpreted data. LW drafted the manuscript. MG-d-A revised manuscript. All authors extensively reviewed the manuscript.

### Conflict of Interest Statement

MG-d-A has a PRMT5 inhibitor patent pending and is a PRMT5 inhibitor inventor on a licensing deal with Prelude Therapeutics. The remaining authors declare that the research was conducted in the absence of any commercial or financial relationships that could be construed as a potential conflict of interest.

## Supplementary Material

Supplemental Figure 1 miRNA expression in naive Th cells. **(A)** Cos-7 cells were transfected with empty luciferase plasmid (without *Prmt5* 3′UTR) and indicated miRNAs. Data are expressed as a relative ratio of firefly luciferase to renilla luciferase activity. Data are pooled from 3 independent experiments. **(B–G)** Naive Th cells were activated with anti-CD3/CD28 and *miR-15a* **(B)**, *miR-16* **(C)**, *miR-96* **(D)**, *miR-146a* **(E)**, *miR-146b* **(F)**, and *miR-195* **(G)** expression was monitored by real time PCR. Data are representative of five independent experiments. ^*^*p* < 0.05, ^**^*p* < 0.01, ^***^*p* < 0.001, ^****^*p* < 0.0001.

Supplemental Figure 2 NF-κB/MYC/mTOR axis does not regulate *Prmt5* transcript or *Prmt5*-targeting miRNA expression in murine memory Th cells. **(A–F)** Memory murine MBP TcR Tg Th1 were activated anti-CD3/CD28 for 8 h in the presence of NF-κB inhibitor Bay11 (NF-κBi) **(A,B)**, MYC inhibitor 10058-F4 (MYCi) **(C,D)**, or mTOR inhibitor rapamycin (mTORi) **(E,F)**, and *Prmt5* **(A,C,E)** and *Myc* **(B,D,F)** mRNA expression was measured by real time PCR. Data are representative of three independent experiments. Error bars indicate Mean ± *SD*. Memory murine MBP TcR Tg Th1 **(G–L)** and Th2 **(M–R)** cells were activated on anti-CD3/CD28 and non-validated miRNA *miR-96* **(G,M)**, *miR-15a* **(H,N)**, *miR-16* **(I,O)**, *miR-146a* **(J,P)**, *miR-146b* **(K,Q)**, and *miR-195* **(L,R)** expression was analyzed by real time PCR. Data are representative of five independent experiments. Error bars indicate Mean±SEM. **(S–X)** Memory murine MBP TcR Tg Th1 were activated anti-CD3/CD28 for 24 h in the presence of NF-κBi **(S,T)**, MYCi **(U,V)**, or mTORi **(W,X)**, and *miR-140-3p* **(S,U,W)**, and *miR-322/424* **(T,V,X)** expression was measured by real time PCR. Data are representative of three independent experiments. Error bars indicate Mean ± *SD*. One-way ANOVA, followed by Dunnett's multiple comparison test, ^*^*p* < 0.05, ^**^*p* < 0.01, ^***^*p* < 0.001, ^****^*p* < 0.0001.

Supplemental Figure 3 miRNA expression human memory Th1 and Th2 cells. Human memory Th1 **(A–F)** and Th2 **(G–L)** were activated with anti-CD3/CD28 and *miR-96* **(A,G)**, *miR-15a*, **(B,H)**, *miR-15b* **(C,I)**, *miR-195* **(D,J)**, *miR-16* **(E,K)**, and *miR-322/424* **(F,L)** expression was measured by real time PCR. Data are representative of three independent experiments. Error bars indicate SEM. ^*^*p* < 0.05, ^**^*p* < 0.01, ^***^*p* < 0.001.
