# Supplementary figures and images for: NF-κB/mTOR/MYC Axis Drives PRMT5 Protein Induction After T Cell Activation via Transcriptional and Non-transcriptional Mechanisms
Source: Front Immunol. 2019 Mar 19;10:524. doi: 10.3389/fimmu.2019.00524 (PMC6433977; doi:10.3389/fimmu.2019.00524)

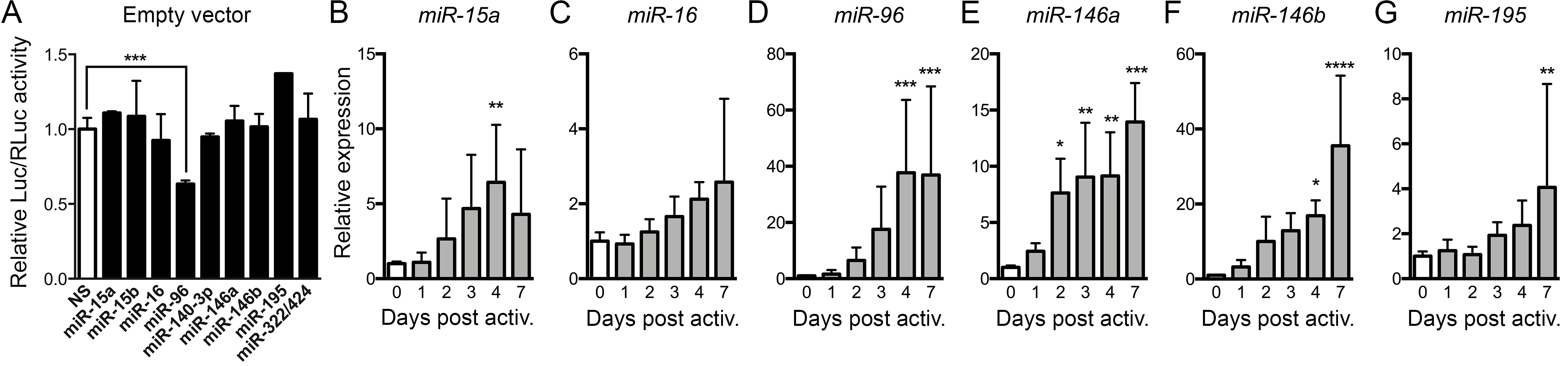

Supplement: Supplemental Figure 1 — miRNA expression in naive Th cells. (A) Cos-7 cells were transfected with empty luciferase plasmid (without Prmt5 3′UTR) and indicated miRNAs. Data are expressed as a relative ratio of firefly luciferase to renilla luciferase activity. Data are pooled from 3 independent experiments. (B–G) Naive Th cells were activated with anti-CD3/CD28 and miR-15a (B), miR-16 (C), miR-96 (D), miR-146a (E), miR-146b (F), and miR-195 (G) expression was monitored by real time PCR. Data are representative of five independent experiments. *p < 0.05, **p < 0.01, ***p < 0.001, ****p < 0.0001. [file Image_1.TIF]

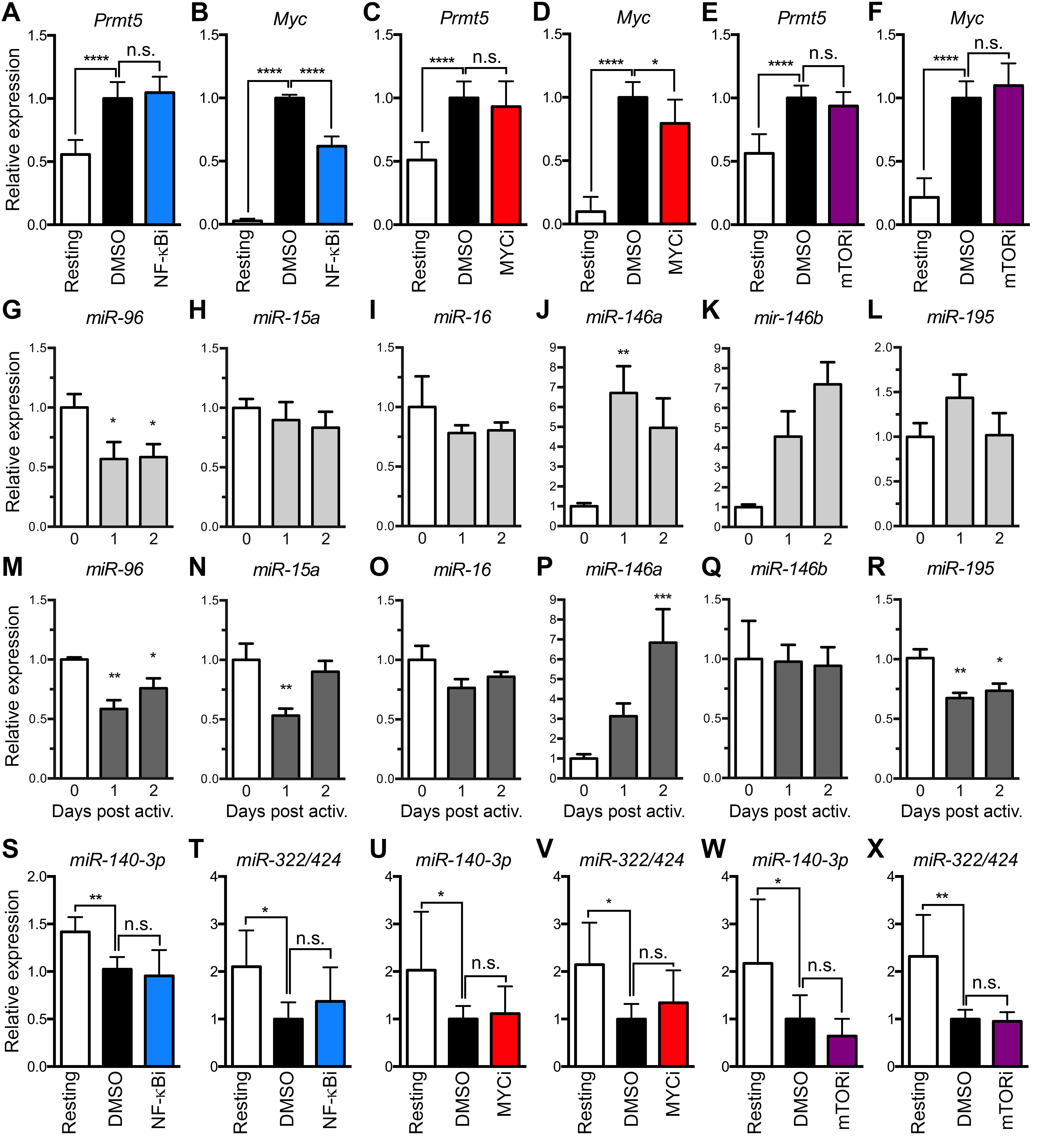

Supplement: Supplemental Figure 2 — NF-κB/MYC/mTOR axis does not regulate Prmt5 transcript or Prmt5-targeting miRNA expression in murine memory Th cells. (A–F) Memory murine MBP TcR Tg Th1 were activated anti-CD3/CD28 for 8 h in the presence of NF-κB inhibitor Bay11 (NF-κBi) (A,B), MYC inhibitor 10058-F4 (MYCi) (C,D), or mTOR inhibitor rapamycin (mTORi) (E,F), and Prmt5 (A,C,E) and Myc (B,D,F) mRNA expression was measured by real time PCR. Data are representative of three independent experiments. Error bars indicate Mean ± SD. Memory murine MBP TcR Tg Th1 (G–L) and Th2 (M–R) cells were activated on anti-CD3/CD28 and non-validated miRNA miR-96 (G,M), miR-15a (H,N), miR-16 (I,O), miR-146a (J,P), miR-146b (K,Q), and miR-195 (L,R) expression was analyzed by real time PCR. Data are representative of five independent experiments. Error bars indicate Mean±SEM. (S–X) Memory murine MBP TcR Tg Th1 were activated anti-CD3/CD28 for 24 h in the presence of NF-κBi (S,T), MYCi (U,V), or mTORi (W,X), and miR-140-3p (S,U,W), and miR-322/424 (T,V,X) expression was measured by real time PCR. Data are representative of three independent experiments. Error bars indicate Mean ± SD. One-way ANOVA, followed by Dunnett's multiple comparison test, *p < 0.05, **p < 0.01, ***p < 0.001, ****p < 0.0001. [file Image_2.TIF]

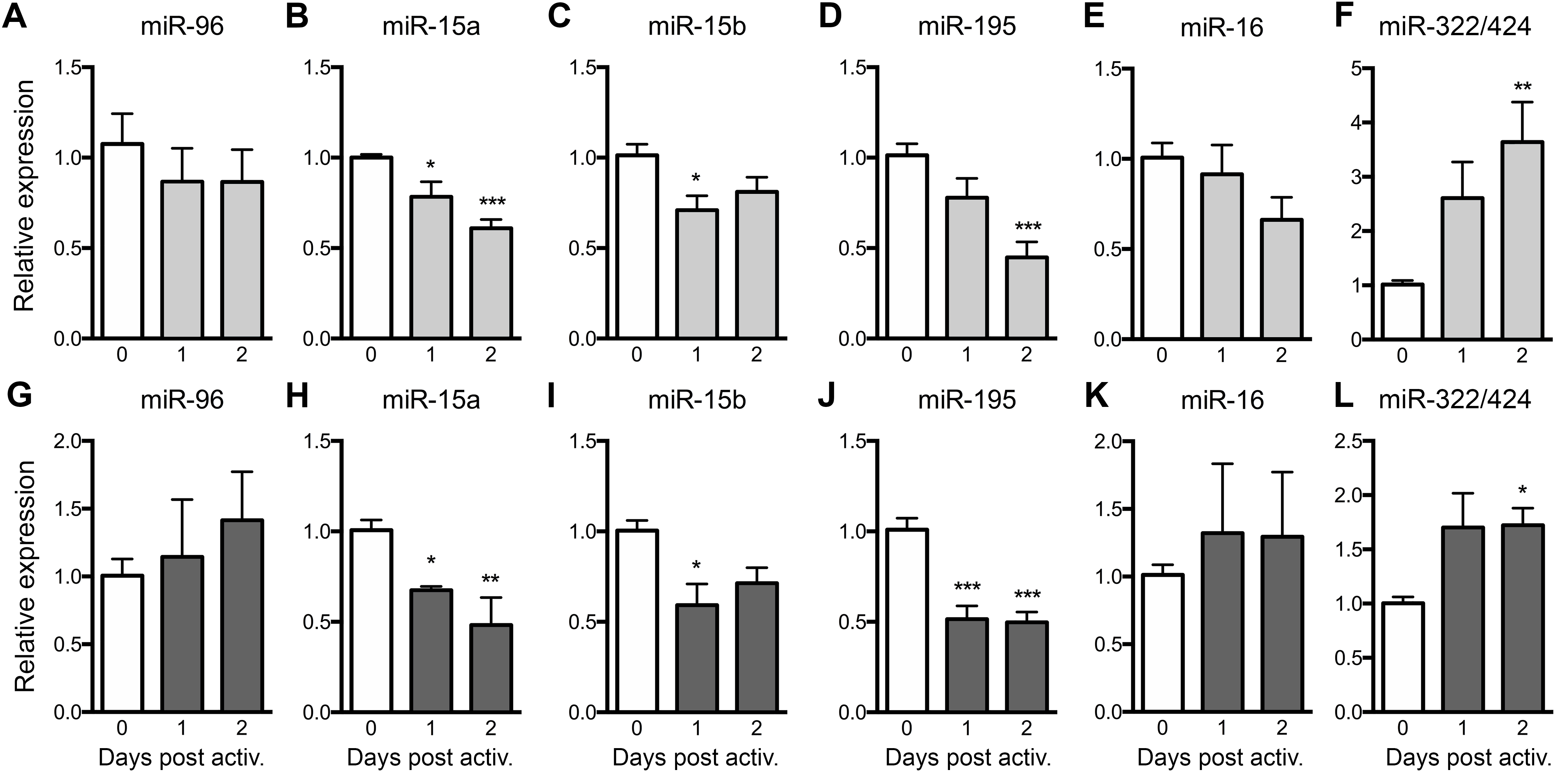

Supplement: Supplemental Figure 3 — miRNA expression human memory Th1 and Th2 cells. Human memory Th1 (A–F) and Th2 (G–L) were activated with anti-CD3/CD28 and miR-96 (A,G), miR-15a, (B,H), miR-15b (C,I), miR-195 (D,J), miR-16 (E,K), and miR-322/424 (F,L) expression was measured by real time PCR. Data are representative of three independent experiments. Error bars indicate SEM. *p < 0.05, **p < 0.01, ***p < 0.001. [file Image_3.TIF]
